# AOP1, a New Live Cell Assay for the Direct and Quantitative Measure of Intracellular Antioxidant Effects

**DOI:** 10.3390/antiox9060471

**Published:** 2020-06-01

**Authors:** Camille Gironde, Mylène Rigal, Cécile Dufour, Christophe Furger

**Affiliations:** AOP/MH2F - LAAS/CNRS, 7 avenue du Colonel Roche, BP 54200, 31031 Toulouse, France; cgironde@laas.fr (C.G.); mrigal@laas.fr (M.R.); cdufour@antioxidant-power.com (C.D.)

**Keywords:** live cell assay, cellular antioxidant effect, radical scavenging, reactive oxygen species (ROS), free radicals, antioxidant assay

## Abstract

Taking advantage of Light Up Cell System (LUCS) technology, which allows for fine monitoring of reactive oxygen species (ROS) production inside live cells, a new assay called Anti Oxidant Power 1 (AOP1) was developed to specifically measure ROS and/or free-radical scavenging effects inside living cells. This method is quantitative and EC_50_s obtained from AOP1 dose-response experiments were determined in order to classify the intracellular antioxidant efficacy of 15 well known antioxidant compounds with different hydrophilic properties. Six of them (epigallocatechin gallate, quercetin, butylated hydroxyanisole (BHA), butylated hydroxytoluene (BHT), ethoxyquin, resveratrol) gave EC_50_s in the range of 7–64 μM, four (Trolox, catechin, epicatechin, EUK134) in the range of 0.14 to 1 mM, and 5 (sulforaphane, astaxanthin, α- and γ-tocopherols, vitamin E acetate) showed only partial or no effect. Interestingly, effects with measurable EC_50_s were observed for compounds with hydrophilic properties (LogP ≤ 5.3), while all antioxidants known to act at the plasma membrane level (LogP ≥ 10.3) had partial or no effect. Sulforaphane, a hydrophilic but strict Keap1/Nrf2 pathway enhancer, did not show any effect either. Importantly, AOP1 assay captures both antioxidant and prooxidant effects. Taken together, these results led us to the conclusion that AOP1 assay measures antioxidant effect of compounds that selectively enter the cell, and act as free radical scavengers in the cytosol and/or nucleus level.

## 1. Introduction

Cell-based compound screening for biological activity not only plays an important role in medical research and drug discovery, but also in nutritional sciences (food, feed and dietary supplements), nutraceutical and cosmetic industries. Quantitative assessment of compound cell efficacy is based on end-point dose-response curves with generation of standard quantitative parameters, such as the efficacy concentrations (EC_10_s, EC_50_s and EC_90_s).

For over three decades, an understanding of disease and aging has been associated with the concept of oxidative stress, which is an imbalance of oxidants and antioxidants, in favor of the oxidants [1,2,3,4]. However, it is also well known that oxidative “eustress” is a fundamental process to maintain health where reactive oxygen species (ROSs), free radicals, and oxidatively modified biomolecules act as signaling molecules involved in physiology. The complexity of oxidative stress in both homeostasis and pathology is still investigated, and the interest of food and pharmaceutical industries towards the discovery of new natural products that can act as antioxidants or, alternatively, prooxidants, is growing.

Different approaches using in vitro conditions, living cells, animal models or human cohorts (for clinical trials) are traditionally used to address antioxidant properties and/or effects. American and European legislations have their respective regulations in regard to safety and toxicity, and to the health claims that could be possibly related to antioxidants and oxidative damage and for which scientific requirements must be provided [5]. Recently, the actual value of classical chemical in vitro antioxidant assays, usually carried out in cell-free context, has been questioned [6], and these assays are now considered inappropriate. In 2012 indeed, the USDA removed the Oxygen Radical Absorbance Capacity (ORAC) values from their website, “due to mounting evidence that the values indicating antioxidant activity have no relevance to the effects of specific bioactive compounds, including polyphenols on human health”. At the same time, animal-based assays became discredited because of the ethical, low throughput and interspecies extrapolation concerns they cause [7]. They were consequently banned in cosmetics regulation from 2013 in the EU [8]. This context opened the door for the generation of more ethical, high-information content, reliable, robust and high-throughput assays, based on living cells [9,10], especially in relevant cell models (cell lines, primary cells, stem cells, organotypic models) now being available.

Up to now, cell-based attempts to study redox activities were mainly based on fluorescence probes that detect ROS activities (i.e., oxidative stress) in the cytosol or the plasma membrane. Genetically encoded biosensors were developed to detect oxidative stress, based on encoded mutants of fluorescent protein (FP)-sensors such as roGFPs, HyPer biosensors and circularly permuted yellow fluorescent proteins [11,12]. A number of redox-sensitive green fluorescent proteins (roGFPs) were created by introducing two cysteine residues, able to form an intramolecular disulfide bond upon equilibration with the intracellular pool of thiols, leading to small structural changes that influence protein fluorescence [13]. Apart from FP approaches, other live cell ROS sensors are C11 BODIPY [14] and malondialdehyde (MDA) for the detection of lipid peroxidation, dihydroethidium (DHE) and MitoSOX [15] for the measure of superoxide production by mitochondria [16], CellROX [17] and 2’, 7’-dichlorofluorescein-diacetate (DCFH-DA) [18] for the detection of cellular ROSs, and monochlorobimane (MCB) for the detection of glutathione depletion [19]. Among them, only the DCFH-DA probe lead to a proper antioxidant cell-based assay (called Cell Antioxidant Assay (CAA)) by using a membrane-based peroxyl radical generator as 2, 2’-azobis (2-amidinopropane) hydrochloride (ABAP) to initiate oxidation. In this model, the DCFH-DA probe is taken up by the cells where it is transformed into DCFH through deacetylation by cellular esterases and trapped within the cells. Oxidation initiated by ABAP at the plasma membrane converts DCFH to its oxidized product DCF, which becomes fluorescent upon excitation. In this model, the measured fluorescence intensity is supposed to be proportional to the level of oxidation initiated by the peroxyl radical generator ABAP. Antioxidants that can scavenge peroxyl radicals in the plasma membrane result in a lower degree of probe oxidation, observed as an attenuated fluorescence level [20]. Even if the DCFH-DA probe has been applied in different contexts in the last decade [21,22,23], its uses are limited by several drawbacks: low signal/noise ratio, autofluorescence of the probe, limitation to detection of antioxidant action on plasma membrane-based lipid peroxidations (as long as ABAP is used) and, above all, its inability to discriminate antioxidant and cytotoxicity effects, which constrains users to add a cell-based cytotoxicity assay, such as MTT, to confirm or infirm the antioxidant effect. In this context, there is an urgent need in both academics and industries for new all-in-one, relevant, easy to implement and robust assays able to demonstrate and, above all, quantify antioxidant effects, such as free radical neutralization, inside living cells.

Here, we report a new universal live-cell assay called Anti Oxidant Power 1 (AOP1), able to measure antioxidant effect inside cells. AOP1 is based on the recent Light-Up Cell System (LUCS) approach, initially developed as a homeostasis assay for cytotoxicity applications [24], and more recently applied as an alternative method to predict human acute oral toxicity [25]. The LUCS homeostasis test is based on the presence of a biosensor and a controlled illumination inside living cells. Briefly, when a fluorescent nucleic acid dye of the asymmetric cyanine family, typically thiazole orange (TO), is added to the cell culture medium, it enters the cells, but is rapidly removed out by membrane-based efflux transport systems (presumably of the MATE family), limiting TO access to nucleic acid targets and resulting in a low fluorescence signal [24]. The application of light (470 nm, 240 mJ. cm^−2^) leads to energy transfer from TO to dioxygen, triggering the generation of ROSs, and, among them, singlet oxygen and radical hydroxyl OH^.^ [24]. This, in turn, evokes a loss of cell homeostasis with an alteration of efflux and/or other cell functions, leading to massive entry of TO and an increase of fluorescence emission. Cell homeostasis status is deduced from the ratio between post- and pre-illumination fluorescence levels.

To address the need for a reliable bioassay that measures intracellular antioxidant activity, we investigated whether intracellular oxidation produced in the course of LUCS process could be controlled by fine tuning of TO concentration and the application of light intensity. We hypothesized that a softened light intensity (470 nm, 24 mJ. cm^−2^) applied in a sequential mode may lead to a weaker generation of reactive species and free radicals, which could be neutralized by antioxidants able to enter the cells and act in the cytosol and/or the nucleus (where the ROSs are produced). Having found the right experimental conditions, we validated the assay by testing (on a dose-response mode) several antioxidant compounds known to exert cell effects, according to various modes of action.

## 2. Materials and Methods

### 2.1. Materials and Reagents

Thiazole orange (TO), resveratrol, Trolox, quercetin, sulforaphane, EUK143, α-tocopherol, γ-tocopherol, vitamin E acetate, butylated hydroxyanisole (BHA), butylated hydroxytoluene (BHT), catechin, epicatechin, epigallocatechin gallate, ethoxyquin, and astaxanthin were purchased from Sigma-Aldrich (Saint-Quentin Fallavier, France). Menadione was a gift from Pharma-Dev, UPS-IRD (Toulouse, France). Gibco DMEM (high glucose, GlutaMAX supplement and pyruvate), fetal bovin serum (FBS) (HyClone), pen-strep solution (100X) (Gibco), 0.05% Trypsin-EDTA (HyClone), Gibco DPBS without Calcium and Magnesium (1X) were purchased from Thermo FisherScientific (Illkirch-Graffenstaden, France). HepG2 (catalog number HB8065), HaCaT (catalog number CRL-2404) and SH-SY5Y (catalog number CRL-2266) cell lines were purchased from the American Type Cell Collection (ATCC) (LGC Standards, Molsheim, France). The Caco-2 cell line was a gift from Led Engineering Development (LED, Montauban, France).

### 2.2. Cell Culture

We used four different classical cell lines representing diverse tissues such as the skin (HaCaT), the central nervous system (neuron-like SH-SY5Y), the liver (HepG2) and the colon (CaCo2). HaCaT (passage 10 to 40), HepG2 (passage 15 to 35) and SH-SY5Y (passage 15 to 35) cells were cultured at 37 °C/5% CO_2_ in GlutaMAX DMEM medium complemented with 10% FBS and 1X pen-strep solution. Caco2 (passage 20 to 35) cells were cultured at 37 °C/5% CO_2_ in GlutaMAX DMEM medium complemented with 20% FBS and 1X pen-strep. Cells were grown up to 70%–80% confluence, then transferred in clear bottom 96-well microplates for 24 h at a density of 10^6^ cells/mL (75μL, 75,000 cells/well) for HepG2 and HaCaT cells, 2.6 × 10^5^ cells/mL (75µL, 20,000 cells/wells) for SH-SY5Y and 4 × 10^5^ cells/mL (75µL, 30,000 cells/wells) for the Caco-2 cells.

### 2.3. AOP1 Assay Experimental Protocol

Stock solutions of compounds/samples were prepared in advance, aliquoted and stored at −20 °C. For the dose-response experiments, nine different concentrations were obtained by serial factor 2 dilutions. For compound/sample preparation, solvent was always ≤ 1% (vol/vol) (4% for astaxanthin and γ-tocopherol) in the highest compound/sample concentration assayed, and maintained at the same proportion during the dilution process. All experiments were carried out in 96-well microplates without using the edge wells. All cell treatments were performed in serum-free medium to avoid interaction with serum components. Each experimental condition was assayed in triplicates, including the solvent control without sample (culture medium, ethanol or DMSO).

Cells were first incubated with compound/sample for 4 h (alternatively 1 h or 24 h for the optimization steps) at 37 °C in 5% CO_2_. TO 4 μM final concentration prepared in serum-free medium (alternatively 0.5, 1 or 2 μM for optimization steps) was added to the cells for 1 h at 37 °C in 5% CO_2_. The fluorescence level was measured (flash number 0) using a Varioskan Flash Spectral Scanning Multimode Reader (Thermo Fisher Scientific, Waltham, MA, USA) set up at 505/535 nm (excitation/emission wavelengths). Microplates were then placed in a dedicated illuminator (24 LEDs, 470 nm, each LED centered on the intersection of 4 wells) (provided by LED Engineering Development, Montauban, France), and illuminated at 24 mJ/cm^2^ (alternatively 12 mJ/cm^2^ for optimization step). Fluorescence level was measured immediately after illumination (flash number 1). The same illumination/reading cycle was repeated at least 20 times.

Two independent experiments were performed for each experimental condition in triplicates. In fluorescence profile figures, error bars correspond to SD values from triplicates. In dose response figures, error bars represent SD from two independent experiments, each in triplicates. In tables, R^2^s represent determination coefficients calculated from sigmoid regression analyses.

### 2.4. Dose Response Post-Analysis

Raw data (relative fluorescence units or RFUs) were further analyzed by Prism8 software (GraphPad, San Diego, CA, USA) to generate dose-response curves. Data were plotted in a kinetics-like mode (light flash number vs. RFU). The result for each experimental condition was normalized to control data (see Section 3.2 in the result part for precisions) and expressed as a cellular antioxidant index (CAI) corresponding to the integration of all normalized data (see Section 3.2 in the result part for precisions).

### 2.5. EC_50_ and Determination Coefficient (R^2^) Evaluation

For dose-response experiments, CAI values were then used to calculate 50% efficacy concentration (EC_50_) values (whenever possible) from a mathematical non-linear regression model (sigmoid fit) given by Prism8, that follows the equation: Y = Bottom + (Top-Bottom)/(1 + 10^((LogEC_50_-X)*HillSlope)), where HillSlope = slope coefficient of the tangent at the inflection point. EC_50_ and R^2^ values were deduced from the regression model.

## 3. Results

### 3.1. AOP1 Assay Optimization

**Figure 1:**
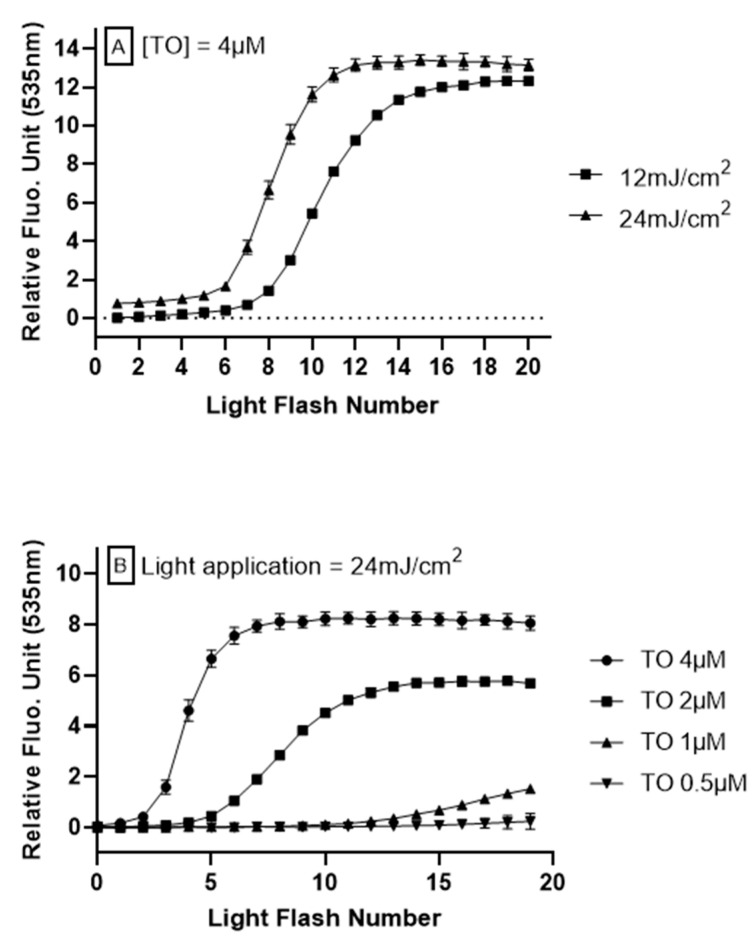
**Anti Oxidant Power 1 (AOP1) assay optimization on HepG2 cells.** Different light energies (470 nm, 12–24 mJ/cm^2^) or thiazole orange (TO) concentrations (0–4 μM, 1 h before light flash) were applied to cells in culture. Fluorescence was measured after each light application. The cycle was repeated 20 times. (**A**) Effect of light exposure with TO concentration fixed at 4 μM. (**B**) Effect of TO concentration with light energy fixed at 24 mJ/cm^2^.

To investigate the relationship between intracellular oxidation generated by TO photoinduction in the LUCS system and light application, HepG2 cells were submitted to varied illuminations at 470 nm, using different exposition protocols. Energy retained in the optimization of LUCS homeostasis assay, 240 mJ/cm^2^ [24], was used as a starting point based on the hypothesis that lower energies would lead to moderate ROS generation, which can be neutralized by exogenous antioxidants. When light is applied at 240 mJ/cm^2^ on a continuous mode at 12 mW/cm^2^ during 20 s, the fluorescence rises up to a plateau that is reached before the end of light application (i.e., <20 s) [24]. Inversely, when light is applied in the range of 12–24 mJ/cm^2^, no fluorescence increase is observed (Figure 1A, light flash number 1). However, repetitions of the same light application lead to a progressive increase in fluorescence level, reaching a plateau. We then optimized the signal (kinetic profile) by modulating two parameters: light energy, which provides excitation energy needed for the photoinduction of TO, and the concentration of TO, which provides the triplet energy needed to activate singlet oxygen production. From different experimental conditions (Figure 1A,B), we kept the kinetic profile obtained when 24 mJ/cm^2^ was applied on HepG2 cells pre-treated for 1 h with TO 4 μM. This concentration was allowed to reach the plateau with the best signal amplitude, and had no cytotoxicity within hours of treatment.

### 3.2. Demonstration of Intracellular Antioxidant Effect Using AOP1 Protocol

We then hypothesized that this kinetic profile can be altered in the presence of antioxidant compounds known to neutralize ROS or free radicals within living cells.

**Figure 2:**
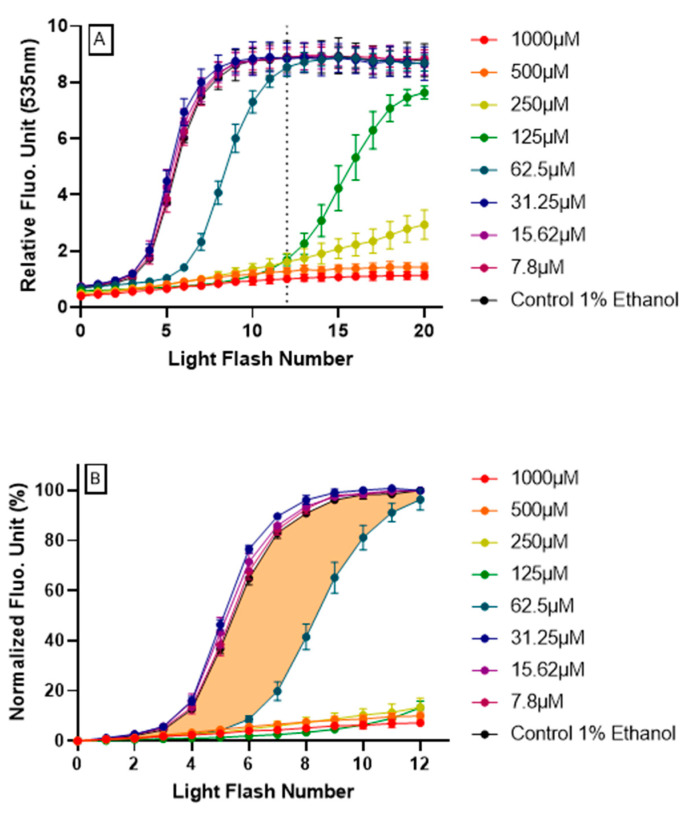
**Resveratrol intracellular antioxidant effect revealed by AOP1 assay on HepG2 cells**.(**A**) Full fluorescence profiles (20 light flashes) obtained for each resveratrol tested concentration (7.8–1000 μM) using the optimized AOP1 protocol (24 mJ/cm^2^, [TO]=4 μM). (**B**) Normalized fluorescence profiles (see text for calculation process); colored surface shows the area under curve (AUC) used for cellular antioxidant index (CAI) estimation (here, the example of resveratrol 62.5 μM).

Human HepG2 cells were treated with increasing doses of resveratrol 4 h, prior to AOP1 procedure. As shown in Figure 2A, higher doses of resveratrol (≥125 μM) totally abolished the fluorescence increase, at least up to light flash number 12, for which fluorescence levels have already reached a plateau for the control condition (no resveratrol). Inversely, lower doses of resveratrol (≤31.25 μM) did not influence control-type AOP1 profile. These results show that resveratrol can abolish (high concentrations) or delay (medium concentrations) the increase of fluorescence produced by photoinduced ROS generation, indicating that resveratrol acts to neutralize intracellular ROSs.

By integrating fluorescence signal as a function of time (or more precisely here, light flash numbers), areas under curves (AUC) calculated from the normalized fluorescence profiles provide a cellular antioxidant index (CAI). CAI was established by a two-step process:

- Firstly, data obtained for the first 12 light flashes needed to reach the plateau in control conditions were normalized using the following equation: NFU% = [(RFU_FNx_ − RFU_FN0_)/(RFU_FN12_ − RFU_FN0_)] × 100 with RFU = relative fluorescent unit, FNx = flash number x and NFU% = normalized fluorescence unit. Normalized profiles are depicted in Figure 2B.
- Secondly, the CAI was calculated by integrating all NFU values following the equation CAI = 1000 − 1000 × (AUC_x_/AUC_control_) where AUC_x_ = _0_∫^12^ NFU_FNx_ and AUC_control_ = _0_∫^12^ NFU_FNcontrol_. An AUC describing the CAI is given in Figure 2B (orange surface) in the case of resveratrol 62.5 μM treatment.

**Figure 3:**
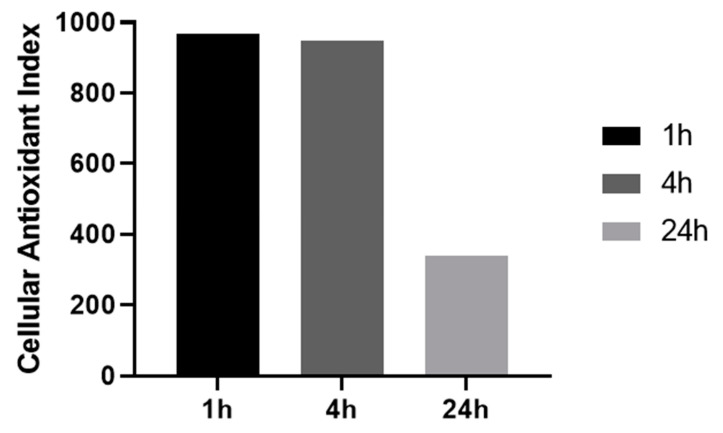
**Effect of antioxidant pre-treatment time on cellular antioxidant index.** HepG2 cells were pretreated with resveratrol 100 or 125 μM for indicated time.

We then investigated the influence of resveratrol pre-treatment time (1 to 24 h) on the CAI values, using the lowest resveratrol concentration for which a full antioxidant effect was observed (100 or 125 μM). As shown in Figure 3, intracellular antioxidant effect remains very high with CAI values of 968.3 and 949.1 for short time pre-treatments (1 h and 4 h, respectively) but goes down to lower value of 338 when cells are pre-treated for 24 h.

### 3.3. AOP1 Dose-Response Profiles Obtained for 15 Classical Antioxidant Compounds

**Figure 4:**
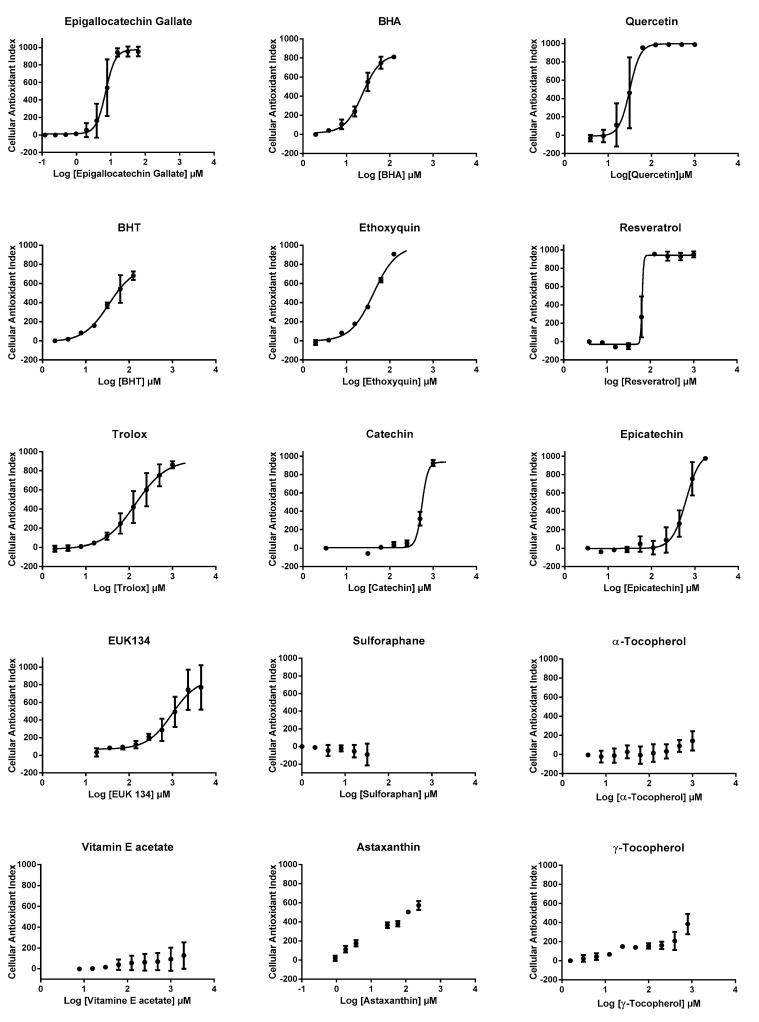
**Dose response profiles obtained with AOP1 assay for 15 classical antioxidant compounds**. All experiments were carried out in triplicate on HepG2 cells and two times, independently. Curves represent a mathematical non-linear regression model (sigmoid fit, see method section for details). Error bars represent SD values of the two independent experiments.

We then used CAI values to establish dose-response profiles from which efficacy concentrations 50% (EC_50_s) were calculated, when possible (Figure 4), after the application of a mathematical non-linear regression model (see method section for details). EC_50_s could be calculated for 10 antioxidants, other compounds presenting either partial or no effect, according to CAI values (Table 1). The highest AOP1 intracellular antioxidant activities (lowest EC_50_ values) were observed for epigallocatechin gallate, quercetin, BHA, BHT, ethoxyquin, resveratrol, Trolox, catechin, epicatechin, EUK134, in this order. We could not determine the EC_50_ for astaxanthin, which only showed a partial effect and for α- and γ-tocopherols, vitamin E acetate and sulforaphane, which did not show any effect. A comparison of EC_50_s with LogP (=LogK_o/w_), the octanol/water partition coefficient which indicates hydrophilic/hydrophobic status of compounds, shows that, apart from sulforaphane, antioxidants with low LogP coefficient (≤5.3) gave a measurable EC_50_ and that antioxidants with higher LogP values (≥10.3) showed only partial (astaxanthin) or no effect (vitamin E family). The case of sulforaphane is interesting. This membrane-permeant compound (LogP = 1.4) is known to exert its antioxidant effect through Keap1-Nfr2 pathway and not as a free radical scavenger, a mode of action that could explain the absence of effect observed using the AOP1 protocol.

### 3.4. Generalization of AOP1 Assay Protocol to Other Human Cell Lines

**Figure 5:**
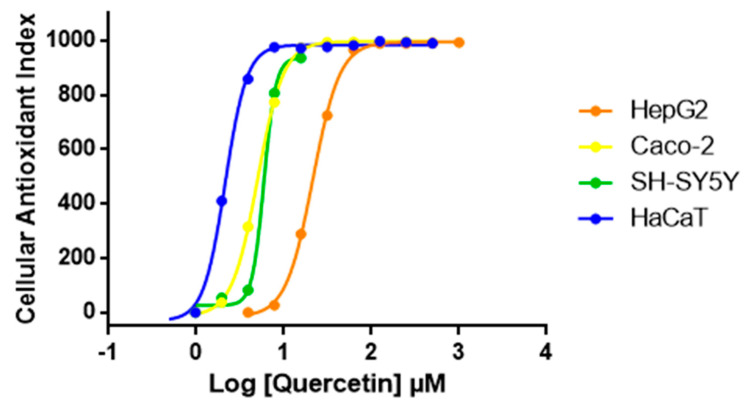
**Dose-response profiles of quercetin obtained with AOP1 assay on various human cell lines.** According to dose-response profiles, HaCaT cells are 10 times more sensitive to quercetin than HepG2 cells.

We then expanded AOP1 protocol optimized in HepG2 to other cell lines. Quercetin was tested with the same AOP1 protocol on various human cell lines. Results presented in Figure 5 show that quercetin EC_50_ varies between cell lines. The compound appears to have highest antioxidant effect in the keratinocyte model (HaCaT) compared to colon (Caco-2), neuron-like (SH-SY5Y) or liver (HepG2) cell models, HaCaT cells (EC_50_ = 2.14 μM), being 10 times more sensitive to quercetin than HepG2 cells (EC_50_ = 23.66 μM) (Table 2).

### 3.5. AOP1 Assay Application to Commercially-Available Products Claiming Antioxidant Properties

**Figure 6:**
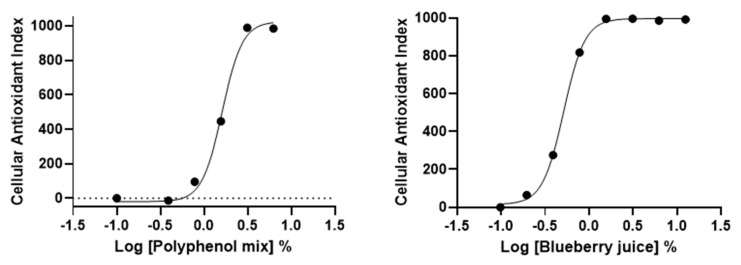
HepG2 dose-response profiles of commercially available beverages obtained with AOP1 assay.

We further applied AOP1 protocol to two commercially available antioxidant-containing beverages, namely a “blueberry juice” and a “polyphenol mix” (containing blackcurrant concentrate, beetroot and green tea extracts and cocoa flavonoids). Dose-response experiments were set using serial dilutions starting from dilution 1/8 (12.5%). Results presented in Figure 6 demonstrate that AOP1 assay can measure intracellular effects of antioxidant-containing commercial products. Both beverages present antioxidant effects with EC_50_ values of 1.62% and 0.52% for the “polyphenol mix” and “blueberry juice”, respectively (Table 3).

### 3.6. Ability of AOP1 Assay to Measure Pro-Oxidative Effects

**Figure 7:**
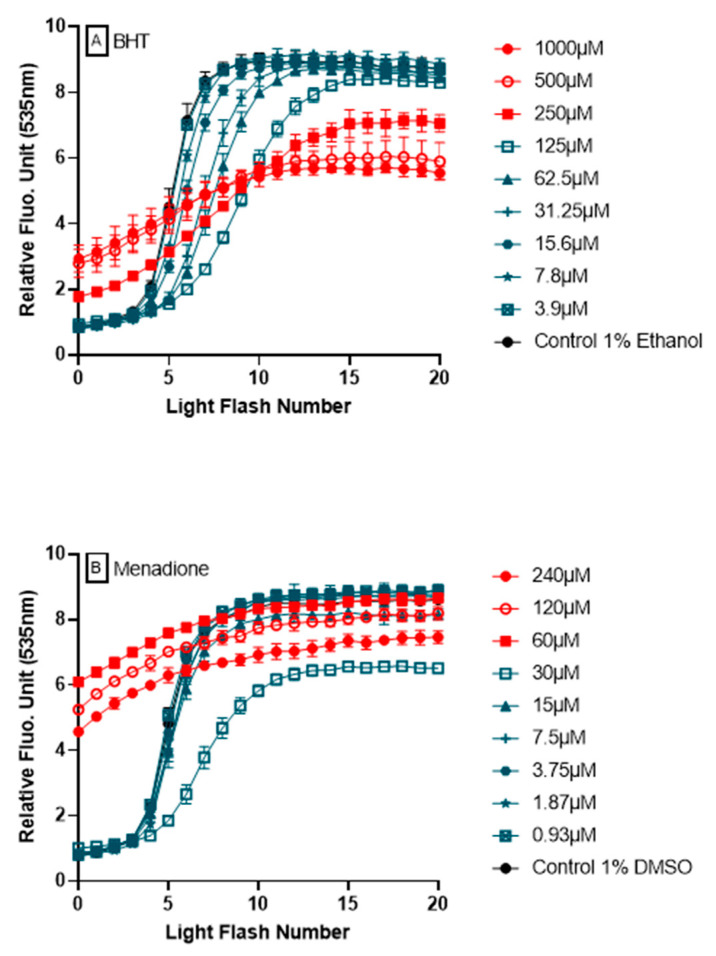
**Detection of pro-oxidant effects by AOP1.** Two examples of compounds, BHT (**A**) and menadione (**B**), showing antioxidant or no effect at low concentrations (in blue) and pro-oxidant effects at high concentrations (in red).

The ability of AOP1 assay to detect pro-oxidative effects from fluorescence profiles is a major attribute and added value to the approach. Figure 7 shows two examples of fluorescence profiles obtained during dose-response experiments using BHT and menadione. In both cases, pro-oxidant effects are observed following their high fluorescence values at T = 0, i.e., after the pretreatment for 4 h, but before the first light flash is applied. These profiles appear to be specific of pro-oxidant effect (see below for argumentation).

## 4. Discussion

The LUCS approach, developed a few years ago as a cell homeostasis assay, was used as a starting point for conceiving AOP1 test. LUCS is based on the discovery that in live cells, light application to the nucleic acid-trapped thiazole orange (TO) fluorescent probe at 470 nm leads to a photoinduction process, followed by an intracellular ROS production. Without light application, fluorescence level remains low because intracellular TO concentration is highly regulated by multidrug efflux transporters. When light is applied, photoinduction occurs and ROS production alters efflux or other cell functions, opening the cell to a massive entry of the probe, ending in an increase of observed fluorescence levels [24]. In the context of LUCS, ROS production has been demonstrated by electronic spin resonance (ESR), using DMPO as a spin trap. We were able to show that photoinduction of TO triggered by high energy illumination (240 mJ/cm^2^) at the cell level provokes the production of (at least) two main actors of the cellular ROS cascade: singlet oxygen and hydroxyl radical. Taking advantage of this knowledge, the initial aim of the present study was to find new experimental conditions that allow to monitor ROS production in live cells in order to develop a quantitative live cell antioxidant assay.

### 4.1. Adaptation of LUCS Protocol to Measure Antioxidant Activity Inside Cells:

In this study, we were able to show that the increase in fluorescence observed in the course of LUCS protocol can be fine-tuned by modulating either light energy or TO concentration. We were surprised to observe that low energy (24 mJ/cm^2^) condition that remains without effect on the fluorescence level eventually evokes, after a few iterations, an increase of fluorescence intensity that leads, on an additive mode, to the plateau already observed after only one intense flash in LUCS assay (Figure 1). We hypothesized that this peculiar profile could be modulated by antioxidant compounds acting inside the cell.

### 4.2. Antioxidants Known as Hydroxyl Radical Scavengers Abolish AOP1 Fluorescence Increase:

Quercetin is a well-known pleiotropic antioxidant acting at the cell level [26]. Notably, it has been demonstrated on human lymphocytes by ESR, using DMPO as a spin trap, that quercetin is a potent hydroxyl radical scavenger [27]. In the present study, we observed that quercetin totally abolishes the fluorescence increase triggered by TO photoinduction (for details, see full kinetic dose-response profile in Supplementary Data). Our dose response study carried out in HepG2 cells (Table 2) gave an EC_50_ of 23.66 μM in accordance with Wilms et al. [27], who found a quercetin scavenging effect in the tens of micromolar range in human lymphocytes. To our knowledge, apart from complicated, onerous and very low throughput technologies (especially when applied to living models), such as ESR, AOP1 assay is the first live cell assay able to measure intracellular free radical scavenging activities.

Catechin, epicatechin and epigallocatechin gallate are part of a family of antioxidants known to scavenge free radicals, such as hydroxyl radical, in the micromolar range in cell free systems [28]. We report here that these three antioxidants exert a full free radical scavenging effect from few to hundreds of micromolar range in HepG2 cells. With an EC_50_ of 7.09 μM, epigallocatechin gallate appeared to be the most efficient antioxidant tested by AOP1, so far (Table 1). This latter result is perfectly in line with epigallocatechin gallate’s radical scavenging data obtained using the ESR approach by Nanjo et al. [28], who established an EC_50_ of 1 μM for hydroxyl radical in a cell free system.

Resveratrol has also been identified as an excellent, although non-specific, radical hydroxyl scavenger in a cell free system [29], where it reacts with OH^.^ radicals by sequential electron proton transfer (SEPT) [30]. Here, we could reveal intracellular antioxidant effect of resveratrol with an EC_50_ of 64.66 μM. Taken together, data obtained with all of these antioxidants demonstrate that AOP1 provides a quantitative measure of free radical scavenging inside cells.

### 4.3. AOP1 Allows to Differentiate Hydrophilic from Hydrophobic Antioxidants:

Apart from the case of sulforaphane which will be discussed below, all tested antioxidants that share some hydrophilic properties (logP ≤ 5.30), and are supposed to enter the cells at least to a certain extent, gave measurable EC_50_s (in the range of 7 to 624 μM), while all the antioxidants sharing hydrophobic properties (logP ≥ 10.30), and known to interact with plasma membrane-based lipids where they break lipidic peroxyl radical chain reactions [31], showed only partial or no effect. This is particularly true with vitamin E components, such as α- and γ-tocopherol, which remained without effect irrespective of the concentration used (up to 1 mM).

Inversely, Trolox, a water-soluble analog of vitamin E developed by exchanging the phytyl side chain with a carboxylate group, and which serves as a standard compound for cell-free assays, such as ORAC or TEAC [32], showed full effect (see Supplementary Data) with an EC_50_ estimated at 138.5 μM. In the context of AOP1, it is particularly interesting to note that Trolox, in contrary to native vitamin E, has been revealed by others to exert a strong hydroxyl radical scavenging activity in a cell-free system [33].

Astaxanthin, a lipid-soluble antioxidant with highly potent peroxyl radical scavenging activities [34,35] was the only lipophilic compound to show some effect but it remained partial (Figure 4), and did not fit with sigmoid model nor allowed to establish an EC_50_ value.

### 4.4. Detection of Dual Pro-Oxidant/Antioxidant Effects by AOP1:

One of the most important features of AOP1 assay is its ability to discriminate antioxidant from prooxidant effects in a unique dose-response experiment. We used menadione, a naphtoquinone also known as vitamin K (allowing carboxylation of glutamyl residues of proteins involved in the blood clotting cascade), with applications in animal feed. Quinones can exert both redox cycling and alkylating activities through two reactions: (1) as prooxidants by reducing oxygen to reactive oxygen species; and (2) as electrophiles by reacting with nucleophilic centers on regulatory molecules to modify them through covalent bond formation [36]. As prooxidant, menadione generates ROSs that oxidize functional groups on signaling proteins (such as protein tyrosine phosphatases), and is used as a generator of oxidative stress or anti-tumor agent [37,38]. Acting as an electrophile, menadione activates Nrf2 pathway through its covalent binding to Keap1 cysteine residues, inducing the expression of Nrf2-target gene products i.e., cytoprotective enzymes such as glutamate-cysteine ligase (GCL) and glutathione S-transferases (GSTs) [39]. Rosen & Freeman [40] also showed that high doses of menadione (100 μM) lead to the generation of superoxide O_2_^−^ and lipid free-radicals in endothelial cells. Here, notably, AOP1 assay captured different biological effects of menadione in a concentration-dependent manner (Figure 7), revealing both a pro-oxidant effect at higher concentrations and an antioxidant or no effect at lower concentrations.

Synthetic antioxidants, such as butylated hydroxyanisole (BHA) and butylated hydroxytoluene (BHT), have been used for decades as potent phenolic compounds in food and cosmetics but their safety and toxicity are still being evaluated [41,42,43,44]. Some studies have reported that both BHT and BHA have tumor-promoting activities, while other ones reported they have anticarcinogenic properties when used at low concentrations [41,45,46]. Ethoxyquin, also used as a food preservative (E324), has seen its authorization suspended by European Commission in June 2017 [47]. Consumer demand has turned to “clean label”, necessitating the search for effective alternative antioxidants from natural sources. When used at low concentrations (1 to 10 μM), ethoxyquin has been reported to show an antioxidant activity (as a protection of human lymphocyte DNA damage by H_2_O_2_ in a comet assay) and no effect at higher concentrations [48]. Upon testing in AOP1 assay, BHA, BHT and ethoxyquin all showed EC_50_s in the range of the tens of micromolar, demonstrating their clear antioxidant activity in HepG2 cells. Interestingly, and in line with the above-mentioned concerns, these three compounds (along with sulforaphane described below) were the only ones among all antioxidants tested in this study to show prooxidative profiles at the higher tested concentrations i.e., ≥125 μM (see Supplementary Data for further details).

Sulforaphane is another compound that showed pro-oxidative effect at concentrations as low as 70 μM, but, unlike other tested compounds sharing this property, it did not exert any AOP1 antioxidant effect. This can be easily interpreted as sulforaphane has been described, like menadione, to physically interact with the Keap1 partner of Nrf2/Keap1 pathway, releasing Nrf2 transcription factor which in turn accumulates in the nucleus and enhances transcription of cytoprotective/antioxidant enzymes such as NAD(P)H quinone reductase (NQO1), GST and others [49,50,51]. Consequently, as a “secondary antioxidant”, it is not surprising that sulforaphane did not show any antioxidant effect in an AOP1 assay.

### 4.5. Benefits of AOP1 Assay:

One of the major benefits of AOP1 assay lies in its dual capacity to detect both antioxidant and prooxidant effects in a single dose-response experiment. This represents a great progress over the DCFH-DA assay (also called CAA assay), which cannot discriminate between antioxidant and cytotoxic effects, and necessitates the addition of a second assay to make sure that observed effects are not due to cytotoxicity.

A low signal-to-noise ratio is one of the other drawbacks of the DCFH-DA assay [52,53], which is circumvented by AOP1. It is usually explained by DCF cell leakage causing extracellular fluorescence in the course of the experiment and limiting interpretation of the results [54]. In comparison, the AOP1 protocol presents a very high signal-to-noise ratio, thanks to the huge increase in quantum yield presented by TO fluorescent probe when it associates with nucleic acids present inside cells. Compared to DCF, TO shows basically no fluorescence in aqueous solution, due to the free rotation of its two rings around their methine bridge. When light is applied, an ultrafast intramolecular twist (100 fs) occurs between the two rings, dissipating the TO relaxation energy by internal conversion instead of photon emission [55].

We can conclude from all these data that the AOP1 protocol provides a new live cell antioxidant method to quantify antioxidant effects, especially for compounds or extracts presenting free radical scavenging activities. Here, we have also demonstrated that the AOP1 protocol works on different cell models, and can be adapted to measure antioxidant effects of complex extracts or products such as commercial beverages. In terms of applications, the assay has been recently used with success to demonstrate and quantify antioxidant effect of a phytocomplex of bilberry (Vaccinium myrtillus) in a standardized 96-well plate format [56].

## Figures and Tables

**Table 1:** AOP1 EC_50_ values obtained for the 15 classical antioxidants presented in Figure 4. LogP (LogK_o/w_) is given as an indicator of the hydrophilic/hydrophobic status of the compound. ND: not determined, because of the lack of an appropriate regression curve.

	EC_50_ (µM)	R^2^	LogP
Epigallocatechin gallate	7.09	0.953	0.00
Quercetin	23.66	0.985	1.48
BHA	31.54	0.941	3.30
BHT	34.25	0.971	5.30
Ethoxyquin	42.06	0.992	3.10
Resveratrol	64.66	0.984	3.10
Trolox	138.50	0.966	2.80
Catechin	555.70	0.990	0.40
Epicatechin	624.70	0.960	0.40
EUK134	979.00	0.877	ND
Sulforaphane	ND	ND	1.40
α-Tocopherol	ND	ND	10.70
Vitamin E acetate	ND	ND	10.30
Astaxanthin	ND	ND	10.30
γ-Tocopherol	ND	ND	10.30

**Table 2:** AOP1 EC50 values obtained for quercetin on various human cell lines presented in Figure 5.

	EC_50_ (µM)	R^2^
HepG2	23.66	0.985
CaCo2	5.02	0.999
SH-SY5Y	5.92	0.998
HaCaT	2.14	0.999

**Table 3:** AOP1 EC50 values obtained for the “polyphenol mix” and “blueberry juice” on HepG2 cells presented in Figure 6.

	EC_50_ (%)	R^2^
Polyphenol Mix	1.62	0.992
Blueberry juice	0.52	0.999

## Supplementary Materials

The following are available online at https://www.mdpi.com/2076-3921/9/6/471/s1, a supplementary figure accompanies this paper.
