# Supplementary material for: AOP1, a New Live Cell Assay for the Direct and Quantitative Measure of Intracellular Antioxidant Effects
Source: Antioxidants (Basel). 2020 Jun 1;9(6):471. doi: 10.3390/antiox9060471 (PMC7346189; doi:10.3390/antiox9060471)
Supplement: Supplementary file 1 [file antioxidants-09-00471-s001.pdf]

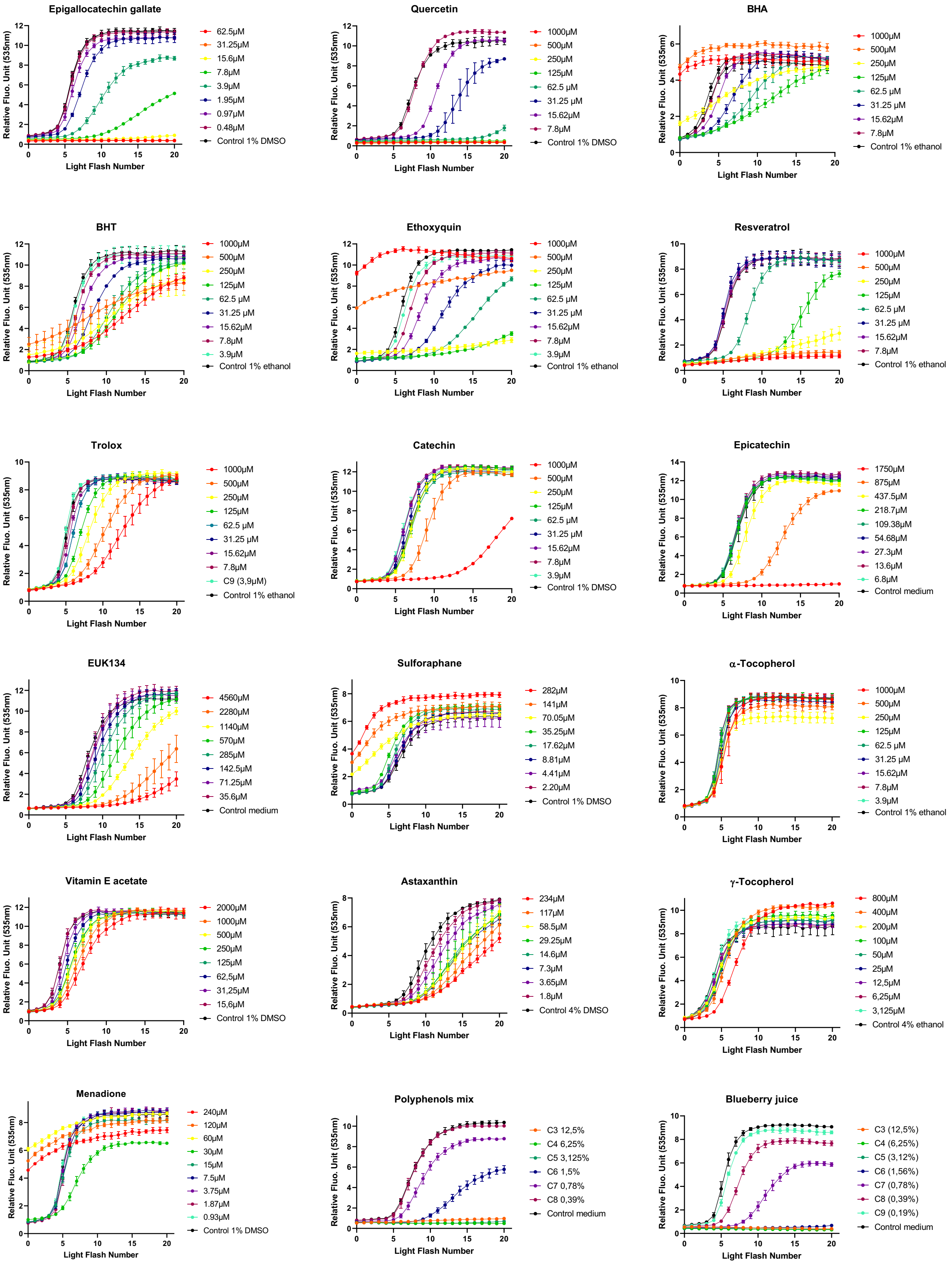

C. Gironde et al – kinetic profiles obtained from dose-response experiments for all the compounds used in the study
